# Problems in measuring the JTC-bias in patients with psychotic disorders with the fish task: a secondary analysis of a baseline assessment of a randomized controlled trial

**DOI:** 10.1186/s12888-020-02959-5

**Published:** 2020-11-23

**Authors:** Nico Pytlik, Daniel Soll, Klaus Hesse, Steffen Moritz, Andreas Bechdolf, Jutta Herrlich, Tilo Kircher, Stefan Klingberg, Martin W. Landsberg, Bernhard W. Müller, Georg Wiedemann, Andreas Wittorf, Wolfgang Wölwer, Michael Wagner, Stephanie Mehl

**Affiliations:** 1 Department of Psychiatry and Psychotherapy & Center for Mind, Brain and Behavior (MCMBB), Philipps-University, Rudolf-Bultmann-Str. 8, 35039 Marburg, Germany; 2 Department of Psychiatry and Psychotherapy, University of Tübingen, Tübingen, Germany; 3 Department of Psychiatry and Psychotherapy, University of Hamburg, Hamburg, Germany; 4 Department of Psychiatry and Psychotherapy, University of Cologne, Cologne, Germany; 5 Department of Psychiatry and Psychotherapy, Vivantes Klinikum am Urban - Teaching Hospital Charité Universitätsmedizin Berlin, Berlin, Germany; 6 Department of Psychiatry, Psychosomatics and Psychotherapy, University of Frankfurt, Frankfurt, Germany; 7 Department of Psychiatry and Psychotherapy, Friedrich-Wilhelms University of Bonn, Bonn, Germany; 8 Department of Psychiatry and Psychotherapy, University of Duisburg-Essen, Essen, Germany; 9 Departmenf of Psychiatry and Psychotherapy, Hospital Fulda, Fulda, Germany; 10 Department of Psychiatry and Psychotherapy, Medical Faculty, Heinrich-Heine University of Düsseldorf, Düsseldorf, Germany

**Keywords:** Schizophrenia, Psychosis, Delusions, Jumping to conclusions, Cognitive bias, Delusional conviction

## Abstract

**Background:**

The jumping to conclusions bias (JTC) is considered to be an important causal factor in theoretical models for the formation and maintenance of delusions. However, recent meta-analytic findings show a rather equivocal pattern of results regarding associations between JTC and delusions. Thus, the present study aims to investigate in a large sample whether the JTC-bias is more pronounced in patients with psychotic disorders in comparison to controls and whether the JTC bias is associated with a more severe delusional conviction, persecutory delusions, and positive symptoms in general.

**Methods:**

Patients with psychotic disorders (*n* = 300) enrolled in a therapy trial and healthy controls (*n* = 51) conducted a variant of the beads task (fish task) as a measure for the JTC-bias at the start of the trial. Further, clinical interviews were used to assess patients’ delusional severity and delusional conviction.

**Results:**

There were no statistically significant differences between patients with psychotic disorders (with 53% displaying the JTC-bias) and controls (41%). Furthermore, there were no statistically significant correlations between JTC measures and persecutory delusions, delusional conviction, and positive symptoms.

**Conclusions:**

We found no differences in JTC between patients with psychotic disorders and healthy controls, which is in part in line with meta-analytic findings using a wide range of JTC task variants. Interestingly, patients with psychotic disorders displayed JTC rates commonly found in the literature, while healthy control subjects showed an unexpectedly high level of JTC. The task variant we used in the present study (fish task) is discussed as a potential reason for our results, as it may induce a more deliberative reasoning style in controls as compared to the traditional beads task. Furthermore, possible implications for the measurement of the JTC-bias, in general, are discussed.

**Trial Registration:**

ISRCTN29242879 (isrctn.com), date of registration: April 12th 2006, retrospectively registered.

## Background

Schizophrenia and psychotic disorders are mental disorders that cause severe and often lasting social-cognitive impairments in most patients [1]. A common feature of psychotic disorders are delusions, which are defined as fixed, false beliefs that usually involve disturbances of the interpretation of experiences [2]. Important factors in the formation and maintenance of delusions are various thinking errors or cognitive biases [3, 4]. Probably the most widely studied bias associated with delusions is the *jumping to conclusions* (JTC) bias [5]. The JTC-bias describes the tendency of individuals to make quick decisions based on what is considered in the literature as little evidence [6].

Traditionally, the JTC-bias is assessed with the beads task [5]. In this task, subjects are presented with two jars containing two types of different colored beads in an opposite ratio (e.g. 85% black, 15% white, and vice versa). The jars will be hidden from view before participants are repeatedly presented with single beads from only one jar. Participants are asked after each presentation if they can decide from which jar the beads are drawn. The number of beads subjects require seeing before making a decision is called *draws to decision* (DTD). Using this experimental setup, healthy subjects usually do not decide after the first or second bead (although the probability for a correct decision after two identical beads already exceeds 94%), but rather ask for more evidence (additional beads) [7–9]. In contrast, patients with psychotic disorders tend to “jump” to a (most often correct) conclusion about the source (jar) already after bead one or two, and this relatively more “risky” epistemological style is termed JTC-bias.

As misunderstanding is a common problem in terms of the traditional beads task [10], a more easily understandable scenario has been employed in the so-called *fish task* [11]: in this task variant, instead of beads which are drawn from two jars, a fisherman fishes different colored fish in one of two ponds. The fish task was found to be feasible in detecting a JTC-bias in individuals with higher subclinical paranoia in comparison to individuals with lower subclinical paranoia [12]. Nevertheless, the first studies that compared patients with psychosis and controls in their JTC-bias with the fish task revealed rather equivocal findings with some studies reporting more pronounced JTC-bias in patients with psychosis in comparison to controls [13] and other studies did not find differences in JTC [14, 15], possibly due to small sample sizes of the studies that prevented them to detect statistically significant group differences.

In general, the JTC-bias is considered to be a fundamental cognitive factor involved in the formation and maintenance of delusion and other positive symptoms of psychotic disorders [4, 16–18]. This impression especially derives from a plethora of studies that indicate a pronounced tendency of a JTC-bias in patients with psychotic disorders or delusion-prone individuals compared to healthy controls, as summarized in two meta-analyses [4, 8, 9]. However, most studies relied solely on the data of delusion-prone healthy individuals instead of patients with a diagnosis of a psychotic disorder [9], which complicates the generalization of the results. Moreover, according to meta-analytic findings, the associations between JTC operationalized as a low number of DTD in the beads task and delusion severity are only of small effect size, with meta-analytic significance tests not being significant [8, 9]. Finally, a recent comparison between meta-analytic evidence and evidence-based on pre-registered studies of the same experimental setup found that meta-analyses systematically overestimate true effects [19].

In the light of the divergent findings, more well-conducted studies with large sample sizes, especially regarding patient samples, are necessary to further clarify the role of the JTC-bias in the formation and maintenance of delusion in patients with psychotic disorders.

One possible explanation for the ambiguous scientific findings is that delusions can be viewed as a multidimensional construct defined by dimensions such as *conviction*, *preoccupation*, *disruption,* and *distress* [20]. Possibly, the JTC-bias is not generally associated with delusions, but rather with specific dimensions of delusions, e.g. the conviction a person presents regarding a delusion. Indeed, several studies suggest that the JTC-bias is related especially to a more pronounced delusional conviction [20–22]. However, given the small number of studies examining the association between the JTC-bias and delusional conviction, these findings should be regarded as preliminary and need further investigation.

Theoretically, the “premature” decision-making [3] in the context of the JTC-bias results in a rapid acceptance of delusional ideas and may consequently contribute to the formation and maintenance of delusional beliefs and positive symptoms in general [4]. However, despite a multitude of studies examining the JTC-bias in psychotic disorders, the topic of different delusional themes (e.g. delusions of persecution) has hardly been researched. This lack of research has been criticized by several authors [12, 23, 24]. Persecutory delusions are the most common delusional theme [25] and most theoretical considerations imply the JTC-bias as an important factor in the formation and maintenance of paranoid ideation and persecutory delusions in particular [23, 26, 27]. Preliminary evidence suggests that interventions targeting reasoning biases like JTC could help to reduce persecutory ideation in patients [28, 29]. This is why we consider it highly relevant to scrutinize not only the possible association of the JTC-bias and positive symptoms in general but also with the specific symptom domain of persecutory delusions.

This study aims to assess the JTC-bias and DTD in a large patient sample with the fish task allowing for a detailed analysis of delusional dimensions and delusional content and their association with the JTC-bias. First, in line with prior studies, we hypothesized that patients with psychotic disorders display a more pronounced JTC-bias and lower DTD score compared to healthy controls (hypothesis 1). Second, we predicted less pronounced DTD to be associated with delusional conviction, persecutory delusions in particular and positive symptoms in general (hypothesis 2).

## Methods

### Subjects

The present study is a secondary analysis of the data of 300 patients with schizophrenia-spectrum disorders and 51 healthy controls who participated in the *cognitive behavioural therapy for persistent positive symptoms (CBTp) in psychosis* trial [30], a multi-centered randomized controlled trial investigating the efficacy of *Cognitive Behavior Therapy for Psychosis* for patients with psychotic disorders compared to supportive therapy. All six centers were equally involved in recruitment and enrolled an equal number of patients. During the recruitment phase, all patients with psychotic disorders in all inpatient facilities of the participating institutions were informed about the study before discharge and screened for their eligibility. Comparable systematic recruitment strategies were carried out mainly in the outpatient facilities of the centers, but also in assisted living facilities and other support services. All patients with a preliminary diagnosis of a psychotic disorder were recruited, unless they met certain “obvious” exclusion criteria (e.g. lack of language skills or substance dependence as a primary problem). In the case of a refusal to participate in the study, the patient was asked to state the reasons for non-participation and to agree with the assessment of symptom severity (PANSS) at this time point. If a patient agreed to participate in the study, more detailed assessments of the inclusion criteria were performed in the course of several appointments, and informed written consent was obtained. All participating patients were randomly assigned to one of the treatment conditions (1:1).

Concerning non-clinical controls, all centers participated equally in their recruitment, which took place via press announcements. Due to the funding restriction, the recruitment plan involved 60 non-clinical controls. Controls were matched with regard to age and gender to the first 60 patients that were enrolled in the study. As the funding was insufficient, recruitment of controls was stopped after enrolling 51 controls.

From the total study sample (*n* = 330), 9 patients dropped out before they were asked to participate. Additional 21 patients did not complete the JTC task and were excluded from the sample. The patients included in the analysis (*n* = 300) were diagnosed with schizophrenia (*n* = 244), schizoaffective disorder (*n* = 37), delusional disorder (*n* = 17) and schizophreniform disorder (*n* = 2) using the *Structured Clinical Interview for DSM-IV (SKID-IV)* [31].

Other inclusion criteria were persistent positive symptoms for at least the last 3 months as indicated by a minimum score of four in item P01 (general delusions; *n* = 263) and/ or in item P03 (hallucinations: *n* = 125; both: *n* = 88) in the *Positive and Negative Syndrome Scale* (PANSS) [32], age between 18 and 59 years, adequate language fluency and an estimated verbal intelligence quotient (IQ) larger than 70 in the German vocabulary IQ test *Mehrfachwahl-Wortschatztest* (MWT-B) [33]. One participant with an IQ-score slightly lower than 70 (68) and data of 12 patients without an IQ-score were also included in the analysis, as these patients’ verbal fluency was sufficient to participate in the JTC-task and both interventions. Exclusion criteria for controls were mental disorders in their lifetime, excluded with the *Structured Clinical Interview for DSM-IV* (SCID-IV) [31].

All participants were informed about the assessment and provided written informed consent. This study was approved by the ethics committee at the six centers’ medical faculties.

A shortened version of the data set (with the purpose of complete anonymization to meet the strict data protection requirements in Germany) can be obtained from the paper’s project page on the OSF (https://osf.io/r3n5q/). In addition, we provide a list of variables that have been used in the analyses, but which will only be made available upon request due to data protection concerns. A more complete data set can be provided by the corresponding author after an individual consultation with our universities’ responsible data protection manager.

### Measures

Psychopathology was assessed with the PANSS, which is the most widely used semi-structured clinical interview for assessing symptom severity in patients with schizophrenia [32, 34]. The scale consists of 30 symptoms commonly reported in schizophrenia that can be subsumed to three subscales (positive and negative scale with 7 symptoms each and general psychopathology scale with 16 symptoms). The symptoms are rated on a 7-point Likert scale ranging from 1 (absent) to 7 (extreme). PANSS rating was performed by raters who received ten training sessions in all PANSS items. Inter-rater reliability (correlation *R*^*2*^) was satisfactory to high (.92 for the PANSS positive scale and .86 for the PANSS negative scale) [30, 35]. As proposed by Wallwork et al. [36], the PANSS structure is better represented by a 20-item, five-factor model with the factors *positive factor, negative factor, disorganized/ concrete factor, excited factor,* and *depressed factor.* In the present study, the proposed *positive factor* was used as a measure for positive symptomatology.

The semi-structured *Psychotic Symptom Rating Scale* (PSYRATS) [37] was used to assess delusional conviction. The scale consists of 7 subscales: *amount of preoccupation*, *duration of preoccupation*, *disruption of daily life*, *conviction, amount of distress,* and *intensity of distress.* Interrater reliability and validity were high in a sample of patients with psychotic disorders [37]. In the present study, the subscale *delusional conviction* was used for further analyses.

The JTC-bias was assessed with a modified version of the *beads task* [5]. Instead of the classical beads task, in which beads are drawn from different jars, a scenario was used that is easier to understand [11]: the *fish task*. In this version of the task, a fisherman fishes different colored fish in one out of two ponds. In the fish task, both fish species (blue or orange) were located in the two ponds (A and B) in reversed ratio, 80 orange to 20 blue fish in pond A, and 20 orange and 80 blue fish in pond B. The fish are drawn successively from only one of the ponds and the subjects are asked whether they can decide after the first fish is drawn if the fish stems from pond A or B. This procedure is repeated until ten fish have been presented to them. A decision after one or two fish is considered as JTC-bias. Also, the number of *draws to decision* (DTD) is recorded: the number of fishes the subjects view until they first decide that the fish stem from one pond. In contrast to the original beads task, in the fish task, subjects are asked to estimate the probability (in percentage) that the fish was selected from pond A or pond B. This measure is called *decision threshold*. To reduce the difficulty of the task and its demands on working memory, the previously fished and the new fish were constantly displayed in the upper screen area. A research assistant was present throughout the experiment and was available for questions of the participants. The research assistant first explained test instructions and checked whether the participant comprehended them correctly. After each presentation of a fish, the assistant explicitly pointed out to the participant that all fish caught so far should be taken into consideration in the decision-making process. In the case of clear misunderstandings, the assistant informed the participant, that all fish were only caught from one of the ponds. In addition, the fish ratio in both lakes was also displayed continuously, with a colored highlighting of the fish distribution on the screen. The complete study material is available on the project page of the study on the open science framework (https://osf.io/r3n5q/).

The fish task has been previously used to study the specificity of the JTC-bias to delusions [14], the contribution of hypersalience to the JTC-bias [13], and the association of task performance and changes in delusions [11] and yielded significant differences between subjects scoring above- and below average in the *Paranoia Checklist* [12, 38]. In the present study, DTD was used as an indicator of JTC, as it is the most reliable JTC measure [7].

### Statistical analysis

Following the *central limit theorem* [39], variables in samples of *n* > 30 can be deemed as normally distributed.

First, patients with psychotic disorders were compared with controls in sociodemographic and clinical variables using Fisher’s exact tests and *t*-Tests. In the case of group differences in specific variables, possible relations to the JTC-bias were analyzed and if they were present, variables were included as covariates in subsequent statistical analyses.

The differences between patients with psychotic disorders and controls in JTC (hypothesis 1) were analyzed using Fisher’s exact test for the JTC-bias and a *t*-Test for DTD. In the case of heterogeneous variances, we used Welch-Tests to compare patients with controls. In the case of significant results, we used Bonferroni-Holm corrections to control for multiple testing. As effect sizes, we calculated the *odds ratio* (OR) for dichotomous variables and Hedges’ *g* for continuous variables.

Using Pearson’s two-tailed correlations, we examined the relationship between the extent of JTC-bias (DTD) and delusional conviction (PSYRATS delusional conviction), positive symptoms in general (PANSS positive factor), as well as persecutory delusions (PANSS P06; hypothesis 2).

Additional summary statistics and plots can be obtained from the paper’s project page on the open science framework (https://osf.io/r3n5q/).

## Results

### Sample characteristics

Table 1 shows the sociodemographic and clinical data of patients with psychotic disorders and non-clinical controls. We did not find any significant statistical differences between the patients and controls in terms of age, gender, or level of education.

**Table 1:** Means, standard deviations, and comparisons of patients and healthy controls regarding sociodemographic and clinical measures

	Patients with psychotic disorders (*n* = 300) n (%)/ M (*SD*)	Healthy controls (*n* = 51) n (%)/ M (*SD*)	Test Statistics
*Sociodemographics*			
Gender (female)	117 (39%)	21 (41%)	*χ2*(1) = .097, *p* = .755
Age (y)	37.94 (*9.68*)	35.65 (*9.30)*	*t*(349) = 1.57, *p* = .117
Verbal IQ	106.16 (*15.08*)	114.88 (*15.37*)	*t*(337) = 3.734, *p* < .001
Duration of illness (y)	15.01 (*9.05*)		
*PANSS*			
PANSS total	64.80 (*11.38*)		
PANSS POS	17.44 (*3.58*)		
PANSS NEG	14.28 (*4.34*)		
PANSS GEN	33.16 (*7.07*)		
PANSS positive factor	11.65 (*2.72*)		
PANSS P01	4.37 (*1.11*)		
PANSS P06	3.48 (*1.48*)		
*PSYRATS*			
PSYRATS DS	2.42 (*0.76*)		
PSYRATS AHS	2.03 (*1.00*)		
PSYRATS Delusional conviction	2.57 (*1.22*)		
*JTC measures*			
JTC-bias present	160 (53%)	21 (41%)	*χ*2(1) = 2.724, *p* = .099
Draws to decision (DTD)	3.10 (*2.70*)	3.49 (*2.48*)	*t*(349) = .973, *p* = .331
Decision threshold	62.06 (*29.02*)	67.66 (*28.58*)	*t*(333) = 1.26, *p* = .209
Subjective probability for Pond A – Draw 1	66.60 (*23.18*)	67.18 (*21.79*)	*t*(349) = .167, *p* = .868
Subjective probability for Pond A – Draw 2	71.30 (*19.39*)	72.82 (*17.87*)	*t*(349) = .523, *p* = .601

Verbal IQ measured with the *MWT-B* Mehrfachwahl-Wortschatz-Intelligenztest-B, a German vocabulary IQ test, *PANSS* positive and negative syndrome scale, *POS* positive symptoms scale, *NEG* negative symptoms scale, *GEN* general psychopathology scale, *P01* delusions scale, *P06* PANSS persecution/ suspiciousness scale, *PSYRATS* psychotic symptom rating scale, *DS* delusions subscale, *AHS* auditory hallucinations subscale, *JTC* jumping to conclusions, *DTD* draws to decision

However, we found group differences in verbal intelligence scores, assessed with the MWT-B [33] between patients with psychotic disorders and healthy controls, who showed a higher verbal intelligence score. As no significant association between verbal intelligence scores and JTC-measures was found (all *p* > .05), no statistical adjustment for verbal intelligence was performed.

### Analysis of differences between patients with psychotic disorders and healthy controls (hypothesis 1)

As depicted in Table 1, we found no statistically significant differences between patients with psychotic disorders and controls regarding the presence of the JTC-bias, with Levene’s test indicating homogenous variances between the groups (*p* = .850). On a descriptive level, patients were more likely to display the JTC-bias with an OR = 1.66, 95% CI [.91, 3.02]. Results showed no significant group differences regarding DTD either, with Hedges’ *g* = 0.15.

### Correlations of DTD with different dimensions of delusions (hypothesis 2)

The results suggest no statistically significant correlation between DTD and PSYRATS delusional conviction (*r* = −.097, *p* = .095). Results showed no correlation of DTD and PANSS positive factor [36] or persecutory ideation (PANSS P06) either, with *r* = −.096, *p* = .096 and *r* = −.095, *p* = .102 respectively.

The correlations between the other JTC-measures (decision threshold and JTC-bias present) and PSYRATS delusional conviction, PANSS positive factor, and PANSS P06 were likewise all statistically non-significant (all *p* > .05).

## Discussion

Unexpectedly, we did not find evidence for group differences between patients with psychotic disorders and healthy controls with regard to the JTC-bias (draws to decision and the presence of the bias). Moreover, the degree of evidence required before reaching a conclusion (i.e. the number of draws to decision) was neither associated with delusional conviction, positive symptoms in general or persecutory delusions in particular.

### Comparison between patients with psychotic disorders and healthy controls in JTC assessed with the fish task

Our results are at odds with meta-analytical findings suggesting a more pronounced JTC and DTD in patients with psychotic disorders when compared to healthy controls [8]. Based on the DTD group differences between patients with psychotic disorders and controls (Hedges *g* = 0.52) reported in the meta-analysis of Dudley et al. [8], the present study had a 96% power to detect such a difference. Thus, the possibility that our results are based on random effects is very unlikely.

The fish task was developed to increase the comprehensibility of the beads task paradigm [11] and has revealed JTC group differences between individuals with lower and higher subclinical paranoia [12]. However, an inspection of the data in Dudley et al. [8] suggests that group differences between patients with psychotic disorders and nonclinical controls are larger for the traditional beads task as compared with the fish task.

It should be taken into account that although patients were preselected based on PANSS P1 delusion item scores/ PANSS P3 hallucination item scores larger than three, in the sample, the mean PANSS total score was quite low (*M* = 64.80, *SD* = 11.38). According to Leucht and colleagues [40], a PANSS total mean score in a patient sample above 57 should be interpreted as “moderately ill”, while a total score of 75 indicates that the patient sample can be viewed as “markedly ill”. It could be argued that the relatively moderate mean score of symptom severity in the patient sample could explain the non-significant group differences, as differences between the patient sample and controls might have been reduced. However, this interpretation seems unlikely, as the proportion of patients who presented the JTC-bias in our study with the fish task is comparable with other studies using the beads task (about 50%) [20, 41, 42], while the proportion of controls in our study who presented the JTC-bias (41%) is about three to four times higher compared to other studies with the beads task [10, 23, 43]. However, other studies with the fish task also found that their control sample showed a less cautious behavior with 44% [15] and 42% [14] of the control sample displaying a JTC bias, and these studies also found no significant JTC differences between psychotic patients and controls. Given these results, it seems more likely that the fish task, while formally similar to the beads task, may induce a generally less cautious decision behavior, particularly in controls.

Thus, while our test power was ideal, our control sample seems to present a pronounced tendency to present a JTC-bias, and consequently, we were unable to detect group differences in JTC, in line with the two other studies that comparably used the fish task. Concluding, it is important to take a close look at the task characteristics in the fish task, in contrast to the traditional beads task.

### Methodological considerations on the fish task

How can we explain the different findings concerning group differences between patients with psychotic disorders and controls in the beads task vs. the fish task?

One explanation of differences between the beads task and the fish task might be that we used a task variant of the fish task designed to simultaneously measure DTD and the decision threshold of the subjects. In this variant, participants are first asked to decide from which original pond the fish they see on the screen were fished. Next, they are additionally asked to estimate the respective probability for the fish to stem from one of the two ponds (this procedure is then repeated ten times). It is possible that the healthy control sample we assessed in the study might have been triggered by the additional probability estimation task to show a “riskier” response behavior [12]. This explanation is supported by the results of the two other studies that used the same variant of the fish task and compared the JTC-bias between patients with psychotic disorders and healthy controls who also did not find statistically significant group differences between both groups [14, 15].

A second explanation of the conflicting results is based on different experimental procedures. On the one hand, in the classical beads task, participants are asked after each presentation of a bead, if they can decide from which jar the beads are drawn. If the participants decide for a jar, the trial is completed. On the other hand, in the fish task, if participants decide that the fish stem from one pond, the trial is continued until ten fish are drawn. Thus, the traditional beads task might result in a more cautious decision-making behavior in healthy subjects, as the decision is definite and subjects are asked only to decide once they are “completely sure” [44] and this might explain the more cautious behavior other groups and we detected in the control group.

### Lack of associations with psychopathology

While the task format may have been responsible for a riskier decision behavior, subjects still differ regarding the evidence required before they reach a decision. We also set out to investigate whether DTD relates to delusional conviction as well as positive symptomatology in general and persecutory delusions in particular. Surprisingly, we did not find a correlation between DTD and delusional conviction in the patient sample. This is not in line with past research, as several studies that used the beads task suggest that a low DTD score is related especially to a more pronounced delusional conviction in patients with psychotic disorders [20, 21] and delusion-prone individuals [20–22]. Nevertheless, our results are in line with the two recent meta-analyses that did not find significant associations between DTD and delusion severity in currently deluded patients with psychotic disorders and delusion-prone individuals [8, 9], also their results are primarily or solely based on studies with the beads task.

Interestingly, studies that used the fish task also did not find significant correlations between DTD and measures of delusion severity in controls [12] and patients with psychotic disorders [14]. Thus, as discussed above, it is important to pay close attention to our task variant, the fish task, to explain the different results.

With regard to associations between the JTC-bias and delusions, results point generally more in the direction that associations are less pronounced than previously assumed and that the importance of the JTC-bias for delusion severity has been overestimated to some degree in the past in theoretical models. Although the present study is not a direct replication of previous studies regarding the measurement of the JTC-bias, it is important to take our results into careful account, especially in light of a large scale study that also found that delusional ideation did not predict DTD (measured with the beads task) [45]. Additionally, meta-analytical results primarily obtained with the beads task [8, 9] based on a large number of studies also point in the same direction.

Interestingly, in the fish task variant other groups and we used, participants first view one (and then two) orange fish and are repeatedly asked whether they can decide if the fish stem from the orange pond. Mathematical analysis reveals probabilities for a correct decision of 80% after the first draw and 94,1% after the second draw for the fish task (using a ratio of 80:20), providing the participants with Bayesian factors of four (first draw) respectively 32 (second draw). Bayesian factors of this size are considered “positive” respectively “strong” evidence in the context of data analysis in research [46, 47], deciding at that stage rather rational than biased.

Since not all decisions after one or two fish should be nominated as “jumping to conclusions”, we stress the importance of an analysis of the probabilities. This could explain the rather “equivocal” findings on associations between JTC and delusions (review of Garety and Freeman [4]) that might depend on the probabilities of a correct decision based on the specific fish/bead sequence and ratio framing the JTC-bias (decision after 1–2 fish/beads) as rather more or less risky/rational.

Furthermore, as misunderstandings of the instructions of the beads task occur more often in patients with psychosis than in healthy controls [10], they could also contribute and enlarge the frequently found group differences between patients and healthy controls. This interpretation is supported by the findings of a large study by Tripoli et al. [48], which found that the association between JTC and psychotic symptoms in patients with psychosis seems to be mediated to a large extent by the IQ of the subjects. Therefore, JTC may be a consequence of more basal cognitive deficits, which may contribute to a misunderstanding of the task. If the fish task is indeed easier to understand in comparison to other task variants, this could be accompanied by a more “rational” response behavior on the part of the patients, which is more in line with the response behavior of non-clinical control subjects. Consequently, the fish task could be a valid measurement method for the JTC bias, which, however, occurs much less frequently in patients with psychosis, if their correct understanding of the task is ensured, as compared to studies using the traditional beads task paradigm that is harder to correctly understand.

It is possible that the decision behavior we measure in the beads task/fish task (whether it is risky or rational) might be still an interesting causal factor involved in the formation and maintenance of symptoms of psychotic disorders. Nevertheless, it might not be causally related to delusions in particular, but rather associated with other symptoms of psychotic disorders such as negative symptoms [49, 50], insight [51] or neurocognitive problems [52] (Interestingly, in an exploratory analysis, we found negative symptoms to be associated with JTC, *r* = −.166, *p* < .05).

This is reminiscent of another cognitive bias, a deficit in *theory of mind*, that first seemed like a vital factor in the formation of delusions but later proved to be rather associated with negative and disorganization symptoms [4]. Moreover, as more and more studies raise doubts about the importance of the JTC-bias for delusions, future studies could also focus on other possible factors in the formation and maintenance of delusions, like emotional processes such as worrying, low self-esteem, or negative affect [4].

In future studies that aim to analyze group differences between patients with psychotic disorders and controls, first, it is important to use a more reliable assessment of JTC, possibly by combining different fish ratios (80:20, 60:40) and different sequences of fish/beads (see Speechley et al. [13] for an analysis of probability estimations for different fish sequences and ratios) and pay close attention to the probability for a correct decision after one or two fish in the task that is used, as it could vary largely and result in either a statistically more premature or a more risky decision. Interestingly, neither the traditional beads task nor the fish task paradigm provides a definite correct decision regarding the required number of beads. It would be interesting to address the question of the optimal decision threshold further in future research, possibly by providing incentives for more cautious decisions and by analyzing ROC-curves of decisions. Future research could also build on variants of the fish task that incentivize optimal levels of data gathering [53], which in turn would allow a clear identification of “premature” decisions. Also, the measurement of the JTC-bias could be performed with different task materials (e.g., emotional salient material) [54], additional measures (e.g. the Cognitive Biases Questionnaire for Psychosis) [55] or new task variants (e.g. the box-task [56] and the “what is this” task [44, 57]).

Furthermore, future studies are well-advised to preregister their hypotheses and analyses, as studies that are not preregistered tend to overestimate effects [19]. Finally, in future meta-analyses on JTC, it would also be interesting to take the probability of a correct decision after one or two fish into account, as it might not always be an “extreme” decision [8], but in the case of the fish task, often a rational decision.

### Strength and limitations

The present study has some notable features. We set out to investigate the JTC-bias in a large, patient sample to fill a gap that became apparent in the meta-analyses mentioned above [8]. Due to the large sample size, the broad variance in positive symptoms, and the measurement of delusions using two interviews, the non-significance of our results should encourage a discussion of the measurement of the JTC-bias in general, which goes beyond the mere criticism of the fish task.

However, the study has some important limitations. First, only hypothesis 2 (association of JTC with delusional symptoms) was preregistered in the trial proposal. The control group was only included in the study design during the study and group comparisons between healthy control subjects and patients were not the main objective of the study, which primarily aimed to compare the effectiveness of CBTp and supportive therapy in treating patients with psychotic symptoms and to analyze putative mediators (e.g. cognitive biases such as the JTC-bias) of change in CBTp. Thus, our results regarding group differences between patients with psychosis and controls are exploratory and should be investigated again in a preregistered design. Furthermore, only a limited budget was available for the assessment of a non-clinical control group, which is why a further criticism of the study might relate to the unequal group size regarding the patient sample and the healthy control subjects. Although unbalanced group sizes may be associated with statistical problems, we would have achieved a power of approximately beta = .99 for absolute differences in JTC rates (based on a JTC rate of 50% in patients and 20% in healthy controls [10, 20, 23, 41, 42]), and .96 for group differences in DTD at the effect sizes reported in the literature [8]. As none of the assumptions for the statistical tests carried out are violated, the results can be considered relatively robust despite the different group sizes [58]. Nevertheless, we have re-examined the results using the non-parametric Mann-Whitney-U test, which also does not show significant group differences in DTD. Furthermore, as discussed before, the task variant with the simultaneous measurement of the decision threshold might be responsible for the non-significance of our results. Additionally, as we did not measure the delusion severity of the healthy subjects, we cannot rule out the possibility that our healthy control sample consisted of delusion-prone individuals who might also display a higher level of JTC-bias, in line with other studies in delusion-prone subjects [59, 60]. While we excluded controls who had a mental disorder in their past/ present, we did not assess delusional ideation in the control group (e.g. with questionnaires like the Peters et al. Delusions Inventory) [61]. Thus, our control sample might be more prone to delusional ideation and consequently, we did not find group differences in JTC.

### Conclusions

We did not find evidence for group differences between patients with psychotic disorders and healthy controls regarding the JTC-bias, as measured by the fish task. Furthermore, the JTC-bias was not associated with delusional conviction, positive symptoms in general or persecutory delusions in particular. Overall, associations between the JTC-bias and delusional conviction and positive symptoms might be smaller than previously assumed. The authors, among whom is one of the developers of the fish task variant used in this trial, conclude by arguing that there is an urgent need to rethink the fish task and perhaps also the traditional beads task paradigm as the standard measure for JTC, especially if it includes the simultaneous measurement of the decision threshold of the subjects. Further, it is important to take into account the probability of a correct decision in the task, as it is not always premature.

## Data Availability

Study materials, additional summary statistics and plots, and a shortened version of the data set (with the purpose of complete anonymization to meet the strict data protection requirements in Germany) can be obtained from the paper’s project page on the OSF (https://osf.io/r3n5q/). A more complete data set can be provided by the corresponding author after an individual consultation with our universities’ responsible data protection manager.

## Abbreviations

CBTp: cognitive behavioural therapy for persistent positive symptoms
DTD: draws to decision
JTC: jumping to conclusions
MWT-B: Mehrfachwahl-Wortschatztest
OR: Odds Ratio
PANSS: Positive and Negative Syndrome Scale
PSYRATS: Psychotic Symptom Rating Scale
